# Higher Cho/NAA Ratio in Postoperative Peritumoral Edema Zone Is Associated With Earlier Recurrence of Glioblastoma

**DOI:** 10.3389/fneur.2020.592155

**Published:** 2020-12-04

**Authors:** Yong Cui, Wei Zeng, Haihui Jiang, Xiaohui Ren, Song Lin, Yanzhu Fan, Yapeng Liu, Jizong Zhao

**Affiliations:** ^1^Department of Neurosurgery, Beijing Tiantan Hospital, Capital Medical University, Beijing, China; ^2^National Clinical Research Center for Neurological Diseases, Center of Brain Tumor, Beijing Institute for Brain Disorders and Beijing Key Laboratory of Brain Tumor, Beijing, China; ^3^Department of Neurosurgery, Beijing Electric Power Hospital, Beijing, China

**Keywords:** glioblastoma, magnetic resonance spectroscopy, choline abnormality, peritumoral edema zone, recurrence, survival

## Abstract

**Objective:** To explore the prognostic significance of metabolic parameters in postoperative peritumoral edema zone (PEZ) of patients with glioblastoma (GBM) based on proton magnetic resonance spectroscopy (MRS).

**Methods:** The postoperative MRS data of 67 patients with GBM from Beijing Tiantan Hospital were retrospectively reviewed. Metabolite ratios including Cho/NAA, Cho/Cr, and NAA/Cr in both postoperative PEZ and contralateral normal brain region were recorded. Log-rank analysis and Cox regression model were used to identify parameters correlated with progression-free survival (PFS) and overall survival (OS).

**Results:** Compared with the contralateral normal brain region, postoperative PEZ showed a lower ratio of NAA/Cr (1.20 ± 0.42 vs. 1.81 ± 0.48, *P* < 0.001), and higher ratios of Cho/Cr and Cho/NAA (1.36 ± 0.44 vs. 1.02 ± 0.27, *P* < 0.001 and 1.32 ± 0.59 vs. 0.57 ± 0.14, *P* < 0.001). Both the ratios of Cho/NAA and NAA/Cr were identified as prognostic factors in univariate analysis (*P* < 0.05), while only Cho/NAA ≥ 1.31 was further confirmed as an independent risk factor for early recurrence in the Cox regression model (*P* < 0.01). According to the factors of MGMT promoter unmethylation, without radiotherapy and Cho/NAA ≥ 1.31, a prognostic scoring scale for GBM was established, which could divide patients into low-risk, moderate-risk, and high-risk groups. There was a significant difference of survival rate between the three groups (*P* < 0.001).

**Conclusions:** Higher Cho/NAA ratio in the postoperative PEZ of GBM predicts earlier recurrence and is associated with poor prognosis. The prognostic scoring scale based on clinical, molecular and metabolic parameters of patients with GBM can help doctors to make more precise prediction of survival time and to adjust therapeutic regimens.

## Introduction

Glioblastoma (GBM) is the most common primary malignant tumor of central nervous system (1). It is charactered by rapid proliferation, strong invasion to normal tissue, and dismal prognosis (2). Surgical resection plays a vital role in the treatment protocol of patients with GBM (3, 4). And the resection degree has been proved to be closely correlated with patient's clinical outcome (4). Although the gross-total resection (GTR) evaluated from contrast-enhanced T1 weighted imaging can be achieved in nowadays (5, 6), the residual T2 or fluid attenuated inversion recovery (FLAIR) abnormal signal region surrounding the surgical cavity is frequently found in routine clinical practice (7, 8). The components of this region are quite complicated and studies committed to addressing this issue are relatively rare (9). It may be a combined consequence of surgical injury, tumor cells invasion, or even demyelination, etc., which cannot be differentiated normally by routine magnetic resonance imaging (MRI) (10–13).

1H magnetic resonance spectroscopy (1H-MRS) is widely used to non-invasively detect the biochemical index and metabolic changes of intracranial lesions, which plays a vital role in making precise diagnosis and predicting the prognosis of patients (14, 15). 1H-MRS can reinforce the diagnostic confidence in the following three situations in which conventional MRI may be of limited significance: (1) the distinction of neoplastic and non-neoplastic intracranial lesions (16, 17); (2) the differentiation between tumor recurrence and radiation necrosis (18); (3) the role in biopsy guidance and radiotherapy planning (19, 20).

Therefore, in this study, the residual T2/FLAIR abnormal signal region surrounding the surgical cavity was defined as postoperative peritumoral edema zone (PEZ), which was the major cause of tumor recurrence of GBM. Our primary objective was to compare the metabolic parameters between postoperative PEZ and contralateral region, and further explore the clinical significance of these properties in predicting tumor recurrence.

## Materials and Methods

### Patients Cohort

A total number of 67 patients with diagnosis of GBM were surgically treated in the Neurosurgery department IV of Beijing Tiantan Hospital from January 2014 to August 2015. All patients underwent routine and enhanced MRI and MRS within 48–72 h after operation. Volumetric calculation of tumor has been fully described in a prior study (3). The extent of resection (EOR) was evaluated by two experienced neuro-radiologists who were blinded to clinical information of patients, according to the following equation: (preoperative tumor volume – postoperative tumor volume) / preoperative tumor volume. The EOR for each patient was classified as either GTR (>99%), near-total resection (NTR) (95–99%), or subtotal resection (STR) (80–95%). All pathological slides were morphologically examined and graded according to the World Health Organization (WHO) Classification of Tumors of the Central Nervous System (21, 22). Once pathological diagnosis was confirmed, all the patients were recommended to receive standard Stupp protocol (23). Briefly, radiotherapy divided into 30 daily fractions of 2 Gy each was delivered to patients within 1 month after operation. Concomitant chemotherapy consisted of temozolomide (TMZ) at a dose of 75 mg/m^2^/d was given during the whole procedure of radiotherapy. After a 4-weeks break, patients would receive six cycles of adjuvant TMZ at a dose of 150–200 mg/m^2^/d for 5 days in every 28 days. Unfortunately, seven patients, in this study, quit the continued treatment after operation for personal reason.

### Acquisition of MRI Data

**Figure 1:**
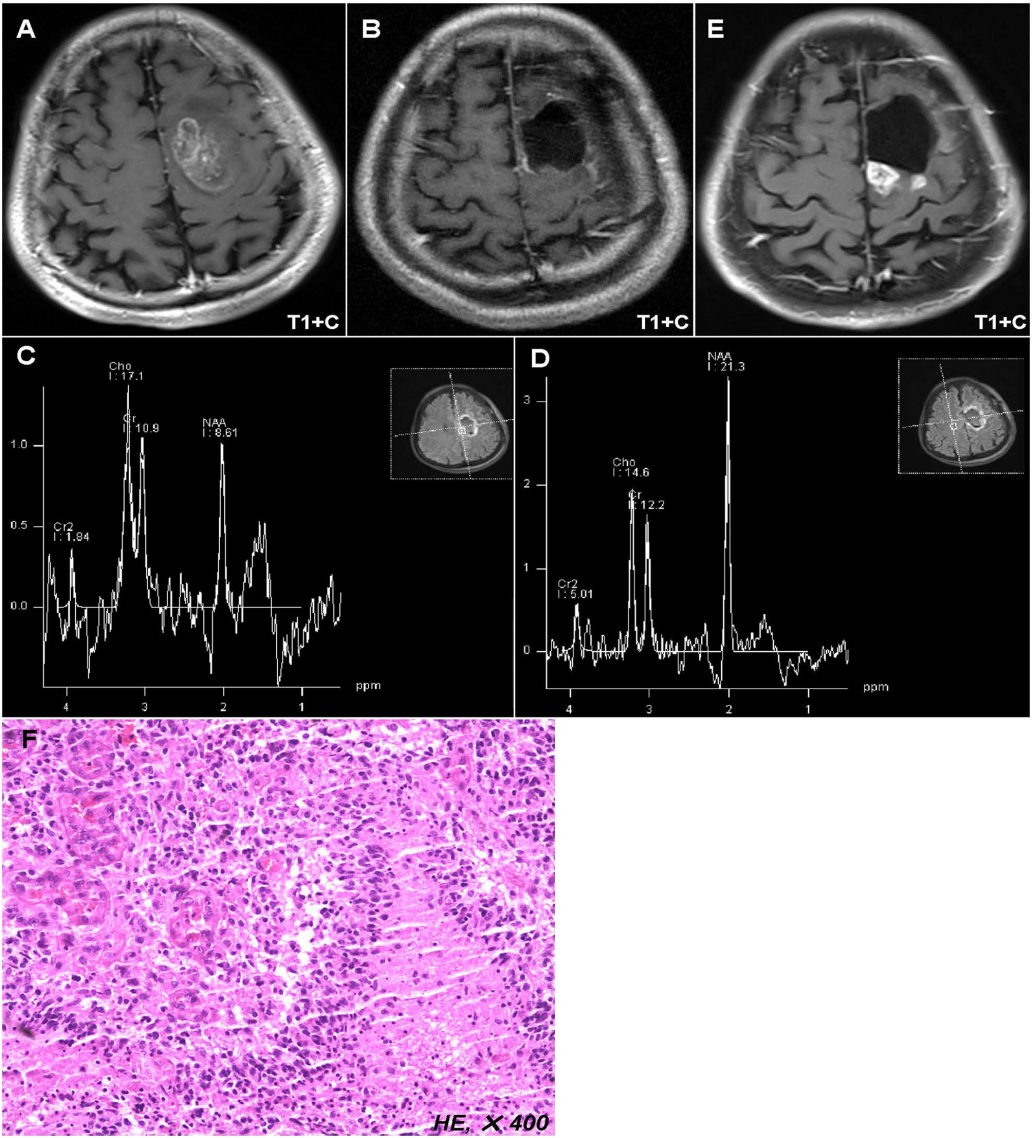
Representative case of this study. Preoperative MR images showed a lesion located in the left anterior central gyrus and supplementary motor area **(A)**. Postoperative MR images displayed that the tumor has been totally removed **(B)**, while the postoperative peritumoral zone showed a higher ratio of Cho/NAA (>1.31) than that in the contralateral region **(C,D)**. Twelve months after operation, the MR images showed a new enhanced lesion in the peritumoral zone **(E)**, which was further confirmed as tumor recurrence by biopsy **(F)**.

All MRI data acquisition was performed on a 3.0 Tesla scanner (Siemens Magnetom Trio Tim, Germany) (Figure 1). The routine sequences included T1-weighted imaging, T2-weighted imaging, and contrast-enhanced imaging. The specific scanning protocol of axial/sagittal T1Flair was field of view (FOV) 240 × 240 mm^2^, matrix 256 × 256, slice thickness 5 mm, spacing 1 mm, time of repetition (TR) 1,500 ms, time of echo (TE) 13 ms, echo train length (ETL) 5, average 1, and scan time of 1 min and 23 s. The specific scanning protocol of Axial T2Flair was FOV 240 × 240 mm^2^, matrix 256 × 180, slice thickness 5 mm, spacing 1 mm, TR 8,000 ms, TE 13 ms, time of inversion (TI) 2,500 ms, ETL 17, average 1, and scan time of 1 min and 48 s. 1H-MRS data was acquired using a 3D MRS sequence with point-resolved spectroscopy (PRESS). Acquisition parameters were as follows: TE 135 ms, TR 1,700 ms, FOV 160 × 160 mm^2^, slice thickness 15 mm, voxel sizes 10 × 10 × 15 mm^3^, average 3, and scan time of 6 min and 53 s. Spectral bandwidth was 2,000 Hz and number of points was 1,024 points. The postoperative PEZ was defined as target region and the contralateral mirror area of target region was served as reference, avoiding bone, subcutaneous fat, hemorrhage, and infarction. Six voxels per case were placed in the target region in order to cover as much of the image abnormality as possible. For cases with residual tumor, at least one voxel was placed on the enhanced region. The relative amounts (area under the curve) of the signals from N-acetyl-aspartate (NAA), choline (Cho), and creatine (Cr) in the target and reference voxels were measured. The MRS detection mentioned above was performed by two independent neuro-radiologists who were blinded to the outcomes of patients. The mean ratios of Cho/NAA, Cho/Cr, NAA/Cr of 12 voxels from two neuro-radiologists was calculated for each patient.

### Follow-Up

Patients were followed up using MRI scans with an interval of 3 months after operation, or 1 month if necessary. Multimodal MR including perfusion, diffusion and MRS was used to rule out radiation necrosis and pseudoprogression. All the patients enrolled in this study would be followed until death. Progression-free survival (PFS) was defined as the time period from the date of operation to date of tumor recurrence or last follow-up. Overall survival (OS) was defined as the time period from the date of operation to the date of death or last follow-up. At the time of data analysis, the median follow-up of this cohort was 44.0 (range: 3.0–58.0) months, and there were 59 (88.1%) patients progressed and 47 (70.1%) patients died.

### Statistical Analysis

Summary of data are presented as the mean ± *SD* for parametric variables and percentage for categorical variables. For the comparison of the metabolic ratios between target and reference regions, paired *t*-test was performed. Receiver operating characteristic (ROC) curves were constructed and were used to determine the area under the curve (AUC) and the optimal cutoff of metabolic ratios in predicting recurrence. Survival as a function of time was plotted using the Kaplan-Meier method, and the Log-rank analysis was used to compare Kaplan-Meier plots. Multivariate proportional hazard regression analysis was used to identify factors associated with PFS and OS. In this analysis, all variables associated with survival in univariate analysis (*P* < 0.05) were included in a step-wise multivariate proportional hazard regression model. All data were analyzed with SPSS software package version 22.0, IBM Corporation, Armonk, NY, USA. Probability values were obtained using 2-sided tests, with statistical significance defined as *P* < 0.05.

## Results

### Overall Characteristics of Study Population

The baseline characteristics of the 67 patients with supratentorial GBM were summarized in Table 1. There were 41 (61.2%) male and 26 (38.8%) female patients with a mean age of 47.1 ± 11.6 years). Of the 67 patients, 23 (34.3%) with tumor located in one lobe, 28 (41.8%) in two lobes, 12 (17.9%) in three lobes, and 4 (6.0%) in four lobes. GTR was achieved in 49 (73.1%) patients, NTR in 13 (19.4%) patients, and STR in 5 (7.5%) patients. Chromosome 1p/19q was intact in all detected cases and only 6 (9.0%) patients harbored isocitrate dehydrogenase (IDH) mutation. Thirty-five (52.2%) patients were identified with O^6^-methylguanine-DNA-methyltransferase (MGMT) promoter methylation.

**Table 1:** Baseline characteristics for all patients with GBM.

**Characteristics**	**No. of patients (%)**
**Age (years)**	
Mean ±*SD*	47.1 ± 11.6
**Gender**	
Male	41 (61.2)
Female	26 (38.8)
**Tumor location**	
1 lobe	23 (34.3)
2 lobes	28 (41.8)
3 lobes	12 (17.9)
4 lobes	4 (6.0)
**Tumor size (cm)**	
Mean ±*SD*	5.1 ± 1.5
**KPS score**	
Median (range)	80 (30–90)
**Extent of resection**	
GTR	49 (73.1)
NTR	13 (19.4)
STR	5 (7.5)
**Chemotherapy**	
Yes	60 (89.6)
**Radiotherapy**	
Yes	60 (89.6)
**1p/19q codeletion**	
Yes	0 (0.0)
**IDH mutation**	
Yes	6 (9.0)
**MGMT methylation**	
Yes	35 (52.2)

*SD, standard deviation; KPS, Karnofsky performance scale; GTR, gross-total resection; NTR, near-total resection; STR, subtotal resection; IDH, isocitrate dehydrogenase; MGMT, O^6^-methylguanine-DNA-methyltransferase*.

### Comparisons of Metabolic Parameters Between PEZ and Contralateral Brain Region

**Figure 2:**
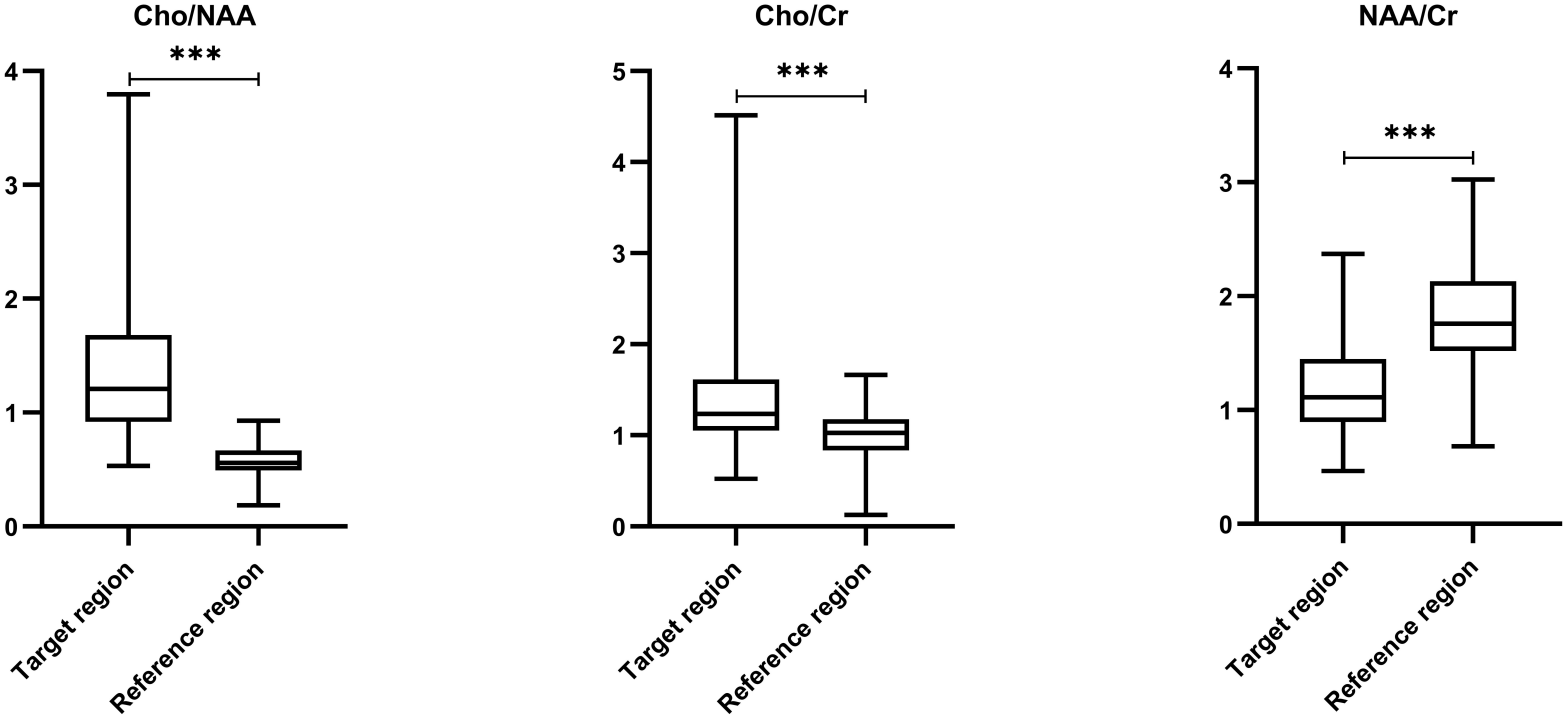
Comparisons of metabolic ratios between target and reference regions by paired *t*-test. Comparing with reference region, ratios of Cho/NAA (0.57 ± 0.14 vs. 1.32 ± 0.59) and Cho/Cr (1.02 ± 0.27 vs. 1.36 ± 0.44) in the target region were remarkably increased, but the ratio of NAA/Cr (1.81 ± 0.48 vs. 1.20 ± 0.42) was significantly decreased (*P* < 0.001). *** *p* < 0.001.

The mean ratios of Cho/NAA, Cho/Cr, and NAA/Cr in the postoperative PEZ (target region) were 1.32 ± 0.59, 1.36 ± 0.44, and 1.20 ± 0.42, respectively; while the mean ratios of Cho/NAA, Cho/Cr and NAA/Cr in the contralateral brain region (reference region) were 0.57 ± 0.14, 1.02 ± 0.27, and 1.81 ± 0.48, respectively (Supplementary Table 1). Compared with the reference region, ratios of Cho/NAA and Cho/Cr in the target region were remarkably increased, but the ratio of NAA/Cr was significantly decreased (*P* < 0.001, Figure 2).

### Values of Metabolic Parameters in Predicting Tumor Recurrence

**Figure 3:**
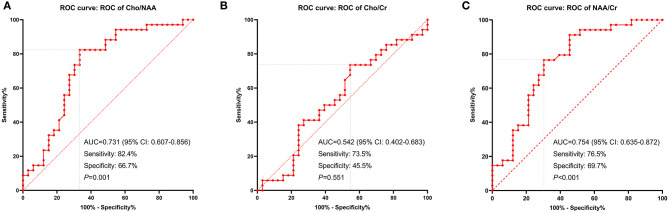
ROC curves displaying the metabolic ratios: Cho/NAA **(A)**, Cho/Cr **(B)**, and NAA/Cr **(C)** in recurrence prediction. The cutoff ratio of Cho/NAA was 1.31 with an area under the curve (AUC) of 0.731 (*P* = 0.001). The cutoff ratio of Cho/Cr was 1.43 with an AUC of 0.542 (*P* = 0.551). The cutoff ratio of NAA/Cr was 1.10 with an AUC of 0.754 (*P* < 0.001).

We used ROC curves to determine the optimal cutoff ratios of Cho/NAA, Cho/Cr, and NAA/Cr in predicting tumor recurrence. As the median PFS of our cohort was 10.0 months, 10-months recurrence was selected to serve as the observation point. According to the results of ROC analyses, the cutoff ratio of Cho/NAA was 1.31 with an AUC of 0.731 (95% confidence interval [CI]: 0.607–0.856, *P* = 0.001). The sensitivity and specificity were 82.4 and 66.7% for recurrence, respectively. The cutoff ratio of Cho/Cr was 1.43 with an AUC of 0.542 (95% CI: 0.402–0.683, *P* = 0.551). The sensitivity and specificity were 73.5 and 45.5% for recurrence, respectively. The cutoff ratio of NAA/Cr was 1.10 with an AUC of 0.754 (95% CI: 0.635–0.872, *P* < 0.001). The sensitivity and specificity were 76.5 and 69.7% for recurrence, respectively (Figure 3).

### Univariate and Multivariate Survival Analysis

Univariate analysis showed that EOR, radiotherapy, chemotherapy, MGMT promoter methylation, and ratios of Cho/NAA and NAA/Cr were correlated with PFS and OS (*P* < 0.05) (Table 2). All these factors correlated with PFS and OS were further enrolled in Cox multivariate analysis. In the Cox proportional hazards regression model, radiotherapy, MGMT promoter methylation and Cho/NAA ratio were confirmed as independent factors associated with both PFS (hazard ratio [HR] = 0.417, 95% CI: 0.174–0.996, *P* = 0.049; HR = 0.401, 95% CI: 0.233–0.690, *P* = 0.001; HR = 2.959, 95% CI: 1.706–5.132, *P* < 0.001, respectively) and OS (HR = 0.083, 95% CI: 0.030–0.230, *P* < 0.001; HR = 0.290, 95% CI: 0.156–0.539, *P* < 0.001; HR = 2.755, 95% CI: 1.512–5.020, *P* = 0.001, respectively) (Table 2).

**Table 2:** Univariate and multivariate survival analysis in all patients.

**Variables**	**Univariate analysis**		***P*-value**	**Multivariate analysis**		***P*-value**
	**HR**	**95% CI**		**HR**	**95% CI**	
**Factors associated with PFS**						
Age (≥50/ <50 years)	0.923	0.544–1.567	0.767			
Gender (male/female)	1.142	0.673–1.938	0.623			
Tumor size (≥4/ <4 cm)	1.765	0.929–3.352	0.082			
KPS score (≥70/ <70)	0.792	0.387–1.621	0.524			
Tumor location (≥3/ <3 lobes)	1.604	0.887–2.898	0.118			
EOR (GTR/No-GTR)	0.464	0.263–0.821	**0.008**			
Radiotherapy (yes/no)	0.326	0.141–0.755	**0.009**	0.417	0.174–0.996	**0.049**
Chemotherapy (yes/no)	0.325	0.142–0.747	**0.008**			
MGMT methylation (yes/no)	0.474	0.280–0.802	**0.005**	0.401	0.233–0.690	**0.001**
IDH mutation (yes/no)	0.925	0.368–2.328	0.869			
Cho/NAA (≥1.31/ <1.31)	2.674	1.578–4.529	** <0.001**	2.959	1.706–5.132	** <0.001**
Cho/Cr (≥1.43/ <1.43)	1.503	0.884–2.554	0.132			
NAA/Cr (≥1.10/ <1.10)	0.379	0.224–0.641	** <0.001**			
**Factors associated with OS**						
Age (≥50/ <50 years)	0.964	0.533–1.742	0.903			
Gender (male/female)	1.041	0.577–1.877	0.894			
Tumor size (≥6/ <6 cm)	2.045	0.951–4.401	0.067			
KPS score (≥70/ <70)	0.919	0.390–2.169	0.847			
Tumor location (≥3/ <3 lobes)	1.731	0.923–3.247	0.087			
EOR (GTR/No-GTR)	0.482	0.258–0.900	**0.022**			
Radiotherapy (yes/no)	0.075	0.027–0.206	** <0.001**	0.083	0.030–0.230	** <0.001**
Chemotherapy (yes/no)	0.249	0.109–0.571	**0.001**			
MGMT methylation (yes/no)	0.311	0.169–0.572	** <0.001**	0.290	0.156–0.539	** <0.001**
IDH mutation (yes/no)	0.510	0.158–1.645	0.260			
Cho/NAA (≥1.31/ <1.31)	2.502	1.396–4.486	**0.002**	2.755	1.512–5.020	**0.001**
Cho/Cr (≥1.43/ <1.43)	1.243	0.689–2.243	0.469			
NAA/Cr (≥1.10/ <1.10)	0.412	0.228–0.746	**0.003**			

*HR, hazard ratio; CI, confidence interval; KPS, Karnofsky performance scale; EOR, extent of resection; GTR, gross-total resection; MGMT, O6-methylguanine-DNA-methyltransferase; IDH, isocitrate dehydrogenase PFS, progression-free survival; OS, overall survival; NAA, N-acetyl-aspartate; Cho, choline; Cr, creatine*.

*Bold value represents p < 0.05*.

### A Proposed Prognostic Scoring Scale for GBM

**Figure 4:**
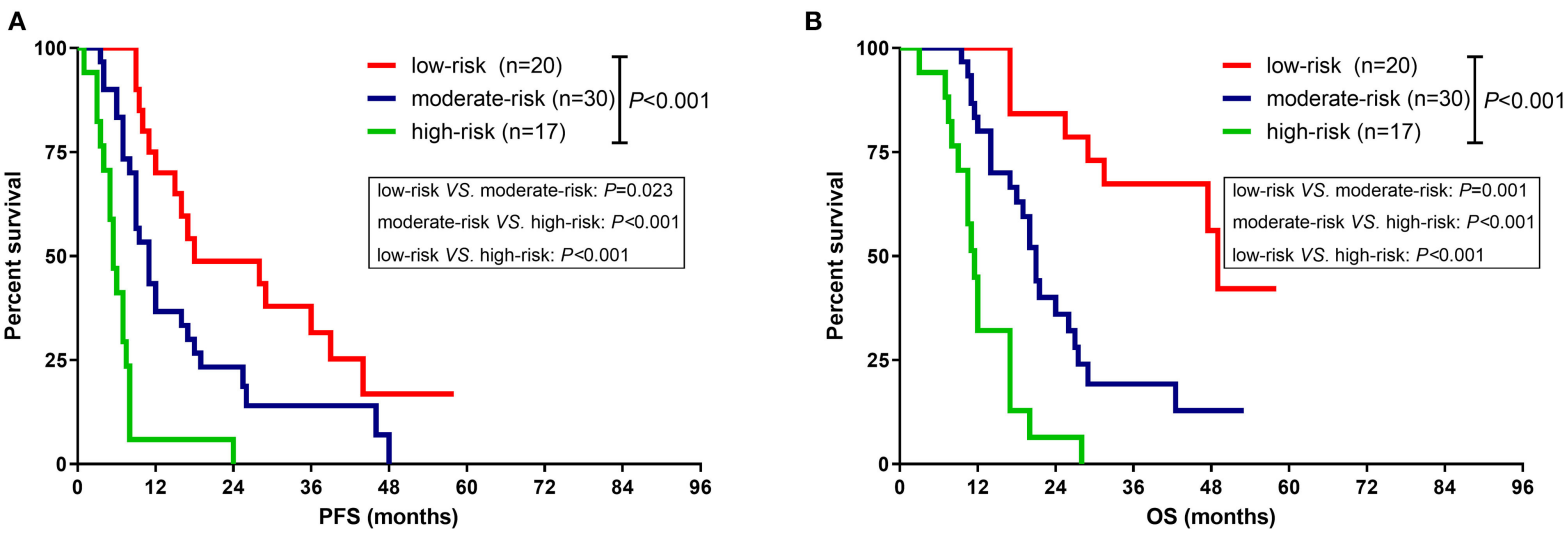
Comparison of survival rate between different subgroups based on the prognostic scoring scale. **(A)** The median PFS for patients in low-risk, moderate-risk and high-risk groups was 18.0, 11.0, and 5.5 months, respectively, which imparted a significant difference (*P* < 0.001). **(B)** The median OS for patients in low-risk, moderate-risk and high-risk groups was 49.0, 21.0, and 11.5 months, respectively, which imparted a significant difference (*P* < 0.001).

According to the independent prognostic factors identified by the multivariate Cox proportional hazards regression model, a prognostic scoring scale was thereby proposed. Briefly, one point was assigned for each of the prognostic risk factors (MGMT promoter unmethylation, without radiotherapy, and Cho/NAA ≥ 1.31). Accordingly, the patients would get a score ranging from 0 to 3 points. Then patients with a score of 0, 1, and equal or more than 2, could be respectively divided into low-risk, moderate-risk, and high-risk subgroups. The median PFS for patients in low-risk, moderate-risk and high-risk groups was 18.0, 11.0, and 5.5 months, respectively, which conferred a significant difference according to the log-rank analysis (*P* < 0.001) (Figure 4A). Meanwhile, a similar survival tendency was also observed in terms of OS among the three subgroups (*P* < 0.001) (Figure 4B).

### Illustrative Case

A 42-years-old male who complained of right limb weakness for 2 months was admitted in our hospital with radiological diagnosis of high-grade glioma. Neurological examination found that the muscle strength of right limbs was grade IV. Radiological result showed a round-like lesion with contrast enhancement in the left anterior central gyrus and supplementary motor area (Figure 1A). The patient underwent surgical resection in our hospital. Postoperative MR implied the enhanced tumor was totally removed (Figure 1B), while the peritumoral zone with abnormal T2/FLAIR signal showed a higher ratio of Cho/NAA (>1.31) than that in the contralateral region (Figures 1C,D). After operation, the patient received concurrent chemoradiotherapy and six cycles adjuvant chemotherapy. Twelve months post of operation, the patient was hospitalized again in our department because of seizures. The MR images showed a new enhanced lesion in the peritumoral zone where initially conferred a higher ratio of Cho/NAA (Figure 1E). To rule out radiation necrosis and pseudoprogression, stereotactic biopsy was performed. And final result confirmed the tumor recurrence (Figure 1F).

## Discussion

GBM, unlike the circumscribed glioma, commonly spreads along the axonal, vascular or subarachnoid space (24). The infiltrated extent of GBM is traditionally refer to the central region on contrast-enhanced T1-weighted images which includes both enhancement and necrosis. However, this central region, to a certain degree, is smaller than the truly infiltrated extent of tumor (25, 26). In contrast, the abnormal region with hyper intensity signal on T2/FLAIR image which reflects a combined consequence of tumor and edema, is larger than the truly infiltrated extent of tumor (27, 28). It's well-established that peritumoral edema is frequently presented in malignant glioma, metastases, and meningioma (29–31). The degree and range of peritumoral edema is correlated with tumor malignancy, and can be used to predict the prognosis of patients (32). Therefore, the peritumoral edema zone (PEZ) is worthy to make further exploration and research.

Given the PEZ was infiltrated with tumor cells which has been validated by histopathological examination, several studies tried to explore the clinical implication of extensively resecting PEZ (3, 33). Li et al., in 2016, conducted a study based on a larger single-center series of GBM patients in order to disclose the association between EOR and prognosis and found that additional resection of PEZ could prolong the survival of patients with GBM (33). Meanwhile, our previous study showed that patients with proliferation-dominant and/or IDH-mutant high-grade astrocytoma could benefit from extensive resection of the FLAIR abnormality region (3). However, current assessment of EOR of patients with GBM is mainly dependent on contrast-enhanced T1-weighted images. Patients who achieved complete removal of the contrast-enhanced region can be defined as gross-total resection. It indicates that the PEZ which is considered as the major origin of tumor recurrence has been remained during operation. How to monitor the change of tumor burden in PEZ is of great significance.

MRS is an imaging technique which can non-invasively detect the metabolism and biochemical changes of tissue. It has been used to differentiate tumor from edema in the PEZ of intracranial malignancies (34, 35). In this study, we systematically analyzed the metabolic changes in postoperative PEZ of patients with GBM. To our knowledge, it's the first study that devoted to disclosing the relationship between metabolic parameters in postoperative PEZ and tumor recurrence. The most important finding was that higher Cho/NAA ratio in postoperative PEZ was associated with early recurrence and the prognostic implication of Cho/NAA has been further confirmed in multivariate analysis. Theoretically, a higher ratio of Cho/NAA implies a more destruction of neuron and a stronger activity of tumor cells (36). This is the reason for higher Cho/NAA ratio in postoperative PEZ harboring poor prognosis. Cordova and colleagues maintained that Cho/NAA could not only identify regions at high risk for contrast-enhancing recurrence, but also show a significant association with PFS in the cohort of GBM patients (37). Tarnawski et al. (38) prospectively analyzed the postoperative tumor bed of 51 patients with malignant gliomas using MRS and found that the ratios of Cho/Cr, Cho/NAA, and myo-inosytol/Cr in PEZ were remarkably increased, but the ratio of NAA/Cr was significantly decreased, which was consistent with our results. They also reported that Lac/NAA > 2 was a strong independent prognostic risk factor (38). In 2015, Tolia et al. explored the prognostic value of MRS metabolites in postoperative irradiated high-grade gliomas and found that Cho/Cr ≥ 2 was associated with earlier recurrence (39). All these results support the hypothesis that substantial tumor cells do exist in PEZ and spectroscopy can provide accurate tumor metabolism information for physicians to better control gliomas.

The second valuable and interesting finding in our study was that patients could be divided into low-risk, moderate-risk, and high-risk subgroups with distinct survival time according to a scoring scale based on the prognostic parameters of radiotherapy, MGMT promoter, and ratio of Cho/NAA. This prognostic scoring scale could lead to an incremental increase in the strength of survival prediction. Li et al. built a survival tree based on older age, larger contrast enhancement, higher ratios of Cho/Cr, Cho/NAA, and lactate/lipid, and lower ratio of Cr/NAA in order to better predict survival (40). But the operation of this survival tree was complicated which limited the use of it in clinical practice. Similarly, TCGA Glioma Phenotype Research Group established a model based on clinical, imaging, and genetic variables which showed the highest accuracy in predicting survival (41). However, the AUC of this predicting model was only 0.679 ± 0.068, which implied a low stability and reliability. In 2019, Gandia-Gonzalez et al. proposed an OS prediction model based on the metabolic markers for patients with glioma, which could do help to make more precise therapeutic decisions (42). Unfortunately, molecular biomarkers have not been included in the model. Therefore, our presented model seems to be relatively stable, easy to use, and more practical.

This study has limitations common to other retrospectively designed studies. First, the sample size is relatively small. However, even given the small sample size, the analytical methods are robust enough to describe the significance of metabolic parameters in predicting tumor recurrence. Second, some patients still fail to reach endpoint event. In the future, we will continue this study until all patients died in order to recheck our results and conclusions. Moreover, several important molecular biomarkers, such as telomerase reverse transcriptase (TERT), phosphate and tension homology deleted on chromosome ten (PTEN), etc., have not been included in our prognostic scoring scale. In spite of these limitations, our study reported some interesting and important findings which might help to extend the application of MRS in the assessment of early recurrence and prognosis prediction of patients with GBM.

## Conclusions

The ratio of Cho/NAA ≥ 1.31 in postoperative PEZ predicts earlier recurrence and is associated with poor prognosis. The prognostic scoring scale based on clinical, molecular and metabolic parameters of patients with GBM can contribute to making more precise prediction of survival time and adjusting therapeutic regimens timely.

## Data Availability Statement

All data supporting the conclusions of this article are available on request from any qualified investigator.

## Ethics Statement

The studies involving human participants were reviewed and approved by Institutional review board of Capital Medical University. The patients/participants provided their written informed consent to participate in this study.

## Author Contributions

YC, WZ, and XR: acquisition of data. YC, WZ, and HJ: analysis and interpretation of data and drafting the article. YC, HJ, and XR: statistical analysis. SL and YC: funding acquisition. YC and JZ: conception and design. JZ: study supervision. All authors contributed to the article and approved the submitted version.

## Conflict of Interest

The authors declare that the research was conducted in the absence of any commercial or financial relationships that could be construed as a potential conflict of interest.
