# Supplementary material for: Higher Cho/NAA Ratio in Postoperative Peritumoral Edema Zone Is Associated With Earlier Recurrence of Glioblastoma
Source: Front Neurol. 2020 Dec 4;11:592155. doi: 10.3389/fneur.2020.592155 (PMC7747764; doi:10.3389/fneur.2020.592155)
Supplement: Supplementary file 1 [file Table_1.doc]

**TABLE S1 Comparison of metabolic parameters between postoperative PEZ and contralateral region.**

| Characteristics | Target region | Reference region | *P* value |
| --- | --- | --- | --- |
| Cho/NAA |  |  | <0.001 |
| Mean±SD | 1.32 ± 0.59 | 0.57 ± 0.14 |  |
| Cho/Cr |  |  | <0.001 |
| Mean±SD | 1.36 ± 0.44 | 1.02 ± 0.27 |  |
| NAA/Cr |  |  | <0.001 |
| Mean±SD | 1.20 ± 0.42 | 1.81 ± 0.48 |  |

Abbreviations: PEZ, peritumoral edema zone; Cho, choline; NAA, N-acetyl-aspartate; Cr, creatine.
